# CATT haplotype of the FKBP5 gene and dissociative phenomenology

**DOI:** 10.1038/s41598-025-13865-9

**Published:** 2025-08-05

**Authors:** Sylvain Moser, Hans Knoblauch, Bertram Müller-Myhsok, Seyma Katrinli, Yara Mekawi, Charles F. Gillespie, Negar Fani, Bekh Bradley, Vasiliki Michopoulos, Jennifer Stevens, Kerry Ressler, Tanja Jovanovic, Alicia K. Smith, Abigail Powers, Stefan Tschöke

**Affiliations:** 1 Research Group Statistical Genetics, Max Planck Institute of Psychiatry, 80804 Munich, Germany; 2 Clinic for Psychiatry and Psychotherapy I (Weissenau), Ulm University, Ulm, Germany; 3 Centre for Psychiatry Suedwuerttemberg, Ravensburg, Germany; 4 Department of Gynecology and Obstetrics, Emory University, Atlanta, GA USA; 5 Department of Psychological and Brain Sciences, University of Louisville, Louisville, KY USA; 6 Department of Psychiatry & Behavioral Sciences, Emory University, Atlanta, GA USA; 7 VA Medical Center, Atlanta, GA USA; 8 Department of Psychiatry, Harvard Medical School, Boston, MA USA; 9 McLean Hospital, Belmont, MA USA; 10 Department of Psychiatry & Behavioral Neuroscience, Wayne State University, Detroit, MI USA; 11 Division of Neuropathology and Neurochemistry, Department of Neurology and Comprehensive Center for Clinical Neurosciences and Mental Health, Medical University of Vienna, Vienna, Austria

**Keywords:** Genetic association study, Genetics research, Psychiatric disorders

## Abstract

Survival mechanisms are evolutionary grown behaviors in life-threatening situations. They are thought to be determined by genetic patterns involved in stress systems, such as the control of the hypothalamic-pituitary-adrenal (HPA) axis. FK506 binding protein 5 (*FKBP5*) is a co-chaperone that is involved in modulating glucocorticoid receptor (GR) sensitivity in response to stress. Dissociation is thought to be one of these survival strategies and appears to be associated with common haplotypes of the *FKBP5* gene formed by four single nucleotide polymorphisms (SNPs) *(rs9296158*,* rs3800373*,* rs1360780*,* and rs9470080)*. The aim of the study was to examine the association between the *FKBP5* haplotypes, type of childhood trauma and different types of dissociative phenomena. Dissociation encompasses a wide range of different phenomena. A common categorization has been made that distinguishes between ‘detachment’ and ‘compartmentalisation’ dissociation. Therefore, both categories were included in the study, including identity dissociation as the most severe form of compartmentalisation dissociation. We analyzed the association between six different types of dissociative phenomena, different types of childhood trauma and the *FKBP5* haplotypes in 194 participants, primarily Black Americans of low socioeconomic status and high trauma burden, who participated in the Grady Trauma Project in Atlanta. We found that only identity dissociation was significantly associated with the CATT FKBP5 haplotype, regardless of the type of childhood trauma. In particular, individuals with one or two CATT haplotypes are 15 times more likely to develop identity dissociation than others. In conclusion, our findings indicate a link between gene variants involved in the regulation of stress systems and self-development under conditions of traumatic stress during the developmental period, which may be important for the study of disorders such as complex post-traumatic stress disorder.

## Introduction

The gene × environment interaction paradigm has been proposed to play a key role in the development of psychological disorders^1^. In this context, the interaction between genetic variants, stress systems, and childhood trauma has been investigated, yielding new insights about how genes interact with the environment to increase risk for psychological symptoms^2^. Dissociative phenomena are common in stress-related psychological disorders and have been conceptualized as an evolutionary grown survival mechanism for inescapable threat^3–6^. But few genetic studies have examined dissociation and, in particular, different dissociative phenomena (for a review, see^7^.

Dissociation is characterized by a disruption or discontinuity of the normal integration of psychological functions^8^. Despite a lack of consensus in defining the concept of dissociation and persistent weaknesses in its operationalization and measurement, a common categorization divides dissociative phenomena into ‘detachment’ and ‘compartmentalisation’ dissociation^9–11^. Detachment includes experiences of separation between the self and environment, i.e. derealisation and depersonalisation. Compartmentalisation is defined as a disruption in the cooperation of mental systems that normally work together, i.e., amnesia, somatisation and identity alteration. Compartmentalisation is also characterised by failure to control emotions, cognitions and behaviour^9,12,13^. The Dissociative Experiences Scale (DES) is the most widely used instrument for studying dissociation in mental disorders^14^, but it covers only a limited range of dissociative phenomena. It has a three-factor structure that assesses derealization/depersonalization, absorption and amnesia^15^. In comparison, the Multiscale Dissociation Inventory (MDI) measures six different dissociative phenomena, covering detachment and compartmentalization dissociation, and a wide range of dissociation severity, with identity dissociation being the most severe form^16^. This phenomenon is thought to be related to childhood abuse during early stages of development^4,17,18^. While detachment dissociation and memory impairment are common in trauma-related disorders regardless of the age at which the trauma occurred^19–21^.

Advances in neuroscience, particularly in neural networks, have expanded our knowledge of dissociative phenomena and shown that they frequently occur in response to stress and appear to have a biological basis^5,22–26^. For example, the dissociative subtype of post-traumatic stress disorder (PTSD) occurs in approximately 40% of individuals with PTSD^27–29^. It is hypothesized that the impaired integration of multisensory inputs may be a trauma-induced disruption of the link between mainly subcortical organized evolutionary survival response systems and higher-order functions, based on phylogenetic and ontogenetic mechanisms.

Based on evolutionary aspects, it can be assumed that survival strategies are determined by genetic and epigenetic patterns involved in stress-systems, e.g., in the control of the hypothalamic-pituitary-adrenal (HPA) axis^30,31^. Dissociation, as an example of such survival strategies, is thought to be moderated by genes and shaped by epigenetic mechanisms underlying environmental adaptation and transgenerational transmission^32–34^. For example, genome-wide association studies have found links between dissociation and genes involved in sensory integration and cognitive processes^35^.

It is therefore surprising that few studies have investigated the genetics of dissociation. Two systematic reviews of biomarkers of pathological dissociation found only fewer than ten studies each, which were difficult to compare due to a lack of overlap in methods and samples^36–38^. Another review of the molecular genetics of dissociative symptomatology included 17 studies and found evidence of an association between dissociative phenomenology and genes related to monoaminergic transmission (5-HTT, COMT), neural plasticity (BDNF), neuropeptide receptors (OXTR), and regulation of the HPA-axis (FKBP5), which have also been associated with other trauma-related symptoms^7^. The reviews emphasize that only a few studies have investigated the relationship between dissociation and genes involved in the HPA-axis^7^. FK506 binding protein 5 (*FKBP5*) is a co-chaperone involved in the HPA-axis and in modulating glucocorticoid receptor (GR) sensitivity in response to stress^1^. Dissociation appears to be associated with common haplotypes of the *FKBP5* gene formed by four single nucleotide polymorphisms (SNPs) (rs9296158, rs3800373, rs1360780, and rs9470080). While one study found an association between chronic childhood maltreatment, the absence of the CATT haplotype of the *FKBP5* gene and the development of dissociative psychopathology in an adolescent population^39^, another study found an association between the presence of the CATT haplotype, childhood maltreatment, and dissociation in a population of high-risk, low-income women^40^. Despite these inconsistencies, a molecular basis for the dissociative phenotype seems evident^7^. This is consistent with the suggestion that the clinical “hyperarousal” or “dissociative” phenotype of PTSD appears to be influenced by polymorphisms of the *FKBP5 gene*^1,30,34^.

To explore the interaction between genes, environment, and dissociation, we investigated *FKBP5* haplotypes and various dissociative phenomena in a high-risk population for traumatic stress. We used a symptom-based approach rather than diagnostic categories^41,42^. We hypothesized that the more severe dissociative phenomena, “compartmentalization” dissociation, are more likely to be associated with the CATT haplotype than “detachment” dissociation, and that these relationships depend on the severity of childhood trauma exposure.

## Methods

### Ethics

All study procedures were approved by the Institutional Review Boards of Emory University School of Medicine and Grady Memorial Hospital and all subjects gave written informed consent to the study.

This study was performed in accordance with the ethical principles outlined in the Declaration of Helsinki and its later amendments. The management of study data conformed to all applicable Health Insurance Portability and Accountability Act rules. All data were de-identified throughout the study to preserve patient anonymity and confidentiality. This post hoc study was conducted under the research exception provisions of the Privacy Rule, 45 CFR 164.514(e), and was exempt from institutional review board informed consent requirements. This study is based on a previously performed and published study and does not contain any new human participants.

### Study participants

A subset of a large-scale study of the impacts of stress- and trauma-related risk factors for PTSD and related behavioral and physical health comorbidities in a high risk, highly trauma exposed urban population of Black Americans in Atlanta, USA^43,44^. They had each experienced at least 1 traumatic event. Individuals were recruited between 2012 and 2015 at Grady Memorial Hospital, Atlanta, Georgia.

### Sample characteristics

The original cohort size was 1026 individuals. This number was reduced to 194 for the SNP analysis due to missing phenotype data. The sample consisted of 23 males with a mean age of 46.35 (SD *±* 12.83) and 171 females with a mean age of 41.43 (SD *±* 11.71). 192 participants self-identified as African American; 1 as Hispanic or Latino; and the race/ethnicity information was missing for 1 participant.

### Psychological assessments

Childhood Trauma Questionnaire (CTQ)^45,46^. The CTQ is a well-validated 28-item questionnaire with five subscales (emotional abuse, emotional neglect, physical abuse, physical neglect, sexual abuse). Its items have five answer choices from 1 (*never true*) to 5 (*very often true*) and some of them are reverse scored. The psychometric properties of the German version of the CTQ are satisfactory and comparable to the American original.

Multiscale Dissociation Inventory (MDI)^16^. The MDI is a 30-item self-report inventory with six subscales to examine six types of dissociation: disengagement, identity dissociation, emotional constriction, memory disturbance, depersonalization, and derealization. Each item is scored on a scale from 1 (*never*) to 5 (*very often*). The scores of the subscales can be converted into T-scores, which allow an empirically based interpretation of the degree of dissociation. Based on the MDI profile form, the following cut-off points of different subscales were used to index dissociation (i.e., binarize these subscales): Disengagement 14 (T-score: 80), Identity Dissociation 9 (T-score 94), Emotional Constriction 13 (T-score 80), Memory Disturbance 11 (T-score 83), Depersonalisation 9 (T-score 82) and Derealisation 11 (T-score 78).

In terms of the category of dissociation, disengagement, emotional constriction, derealisation and depersonalisation were considered to be detachment dissociation, while memory impairment and identity dissociation were considered to be compartmentalisation dissociation^11^.

### Genetic data processing and quality control

#### Genotype quality control

Genotyping is performed by OmniQuad 1 M and OmniExpress arrays. Genotype data processing and quality control (QC) were done as part of the Psychiatric Genomics Consortium PTSD Workgroup (PGC-PTSD) meta-analysis, using standard PGC pipeline RICOPILI, as described in Nievergelt et al., 2019^47^. Briefly, using SNPs with call rates > 95%, samples with (i) call rates < 98%, (ii) deviation from expected inbreeding coefficient (fhet < − 0.2 or > 0.2), or (iii) a discrepancy in reported and estimated sex, as determined by inbreeding coefficients calculated from X chromosome SNPs were removed. Monomorphic SNPs and SNPs with call rates < 98% and a > 2% difference in missing genotypes between PTSD cases and controls were excluded. SNPs with a Hardy-Weinberg equilibrium (HWE) p-value < 1 × 10^−6^ in controls were removed in all samples. Relatedness was calculated using the identity-by-state (IBS) function in PLINK 1.9^48^. In cases where the estimated relatedness (ˆ) between two individuals exceeded 0.2, one of the individuals was excluded from subsequent analysis, while making an effort to keep cases whenever feasible.

#### Global ancestry

To account for possible confounding by common global ancestry in the association between SNPs and phenotype, we computed the first 10 Multi-Dimensional Scaling (MDS) components of the genetic similarity matrix (PC1-PC10) were dataset using plink. The QCed dataset was filtered with a MAF threshold of 0.05, a minimum genotyping frequency of 2% and then LD-pruned with a threshold of 0.2 prior to MDS computation. The 3 first PCs were initially used as covariate in the regression models to account for the global ancestry. As their inclusion did not change the significance of the other predictors, and as they could not be computed for every patient due to missing data, they were not included in the final models in order to not reduce samples size and power.

#### FKBP5 haplotype Estimation

QCed genetic data for the chromosome 6 were first phased using shapeit^49^ and the 1000 Genome Project V3 reference panel (https://mathgen.stats.ox.ac.uk/impute/1000GP_Phase3.tgz) in order to assign each allele to one of the two homologous chromosome. Then for each sample the genotyped allele at the 4 SNPs (rs3800373, rs9296158, rs1360780, and rs9470080) on each of the chromosome were combined, in this order, to form the FKBP5 haplotype.

### Statistical association analysis with single MDI subscales

#### FKBP5 haplotypes

Association analysis between individual MDI subscales and *FKBP5* CATT haplotypes was performed in R using the following linear model (Eq.1) (for continuous MDI subscales, transformed using a rank-based inverse normal transformation) and logistic model (Eq. 2) (for binary MDI subscales), correcting for age, sex and substance abuse:

1 $${\text{Continuous MDI subscale }}\sim {\text{ CATT haplotype }} + {\text{ sex }} + {\text{ age}}$$

2 $${\text{Binary MDI subscale }}\sim {\text{ CATT haplotype }} + {\text{ sex }} + {\text{ age }} + {\text{ substance abuse}}$$

FKBP5 haplotypes and child trauma scales:

Association analysis between individual MDI subscales and *FKBP5* CATT haplotypes x Childhood Trauma and their interaction was performed in R using the following linear model (Eq. 3) (for continuous MDI subscales, transformed using a rank-based inverse normal transformation) and logistic model (Eq. 4) (for binary MDI subscales) correcting for age and sex and substance abuse:

3 $${\text{Continuous MDI subscale }}\sim {\text{ CATT haplotype }} + {\text{ sex }} + {\text{ age}}$$

4 $${\text{Binary MDI subscale }}\sim {\text{ CATT haplotype }} + {\text{ sex }} + {\text{ age}}$$

Relative risk computation:

The Relative Risk, which can be interpreted as the probability of an event occurring in the exposed group (i.e., individuals with the CATT haplotype here) divided by the probability of this event occurring in the non-exposed group, was computed using the following formula:

RR = OR/(1 − Pref)+(Pref∗OR) where Pref is the prevalence in the non-exposed group.

## Results

### Association between *FKBP5* CATT haplotype and identity dissociation

**Fig. 1:**
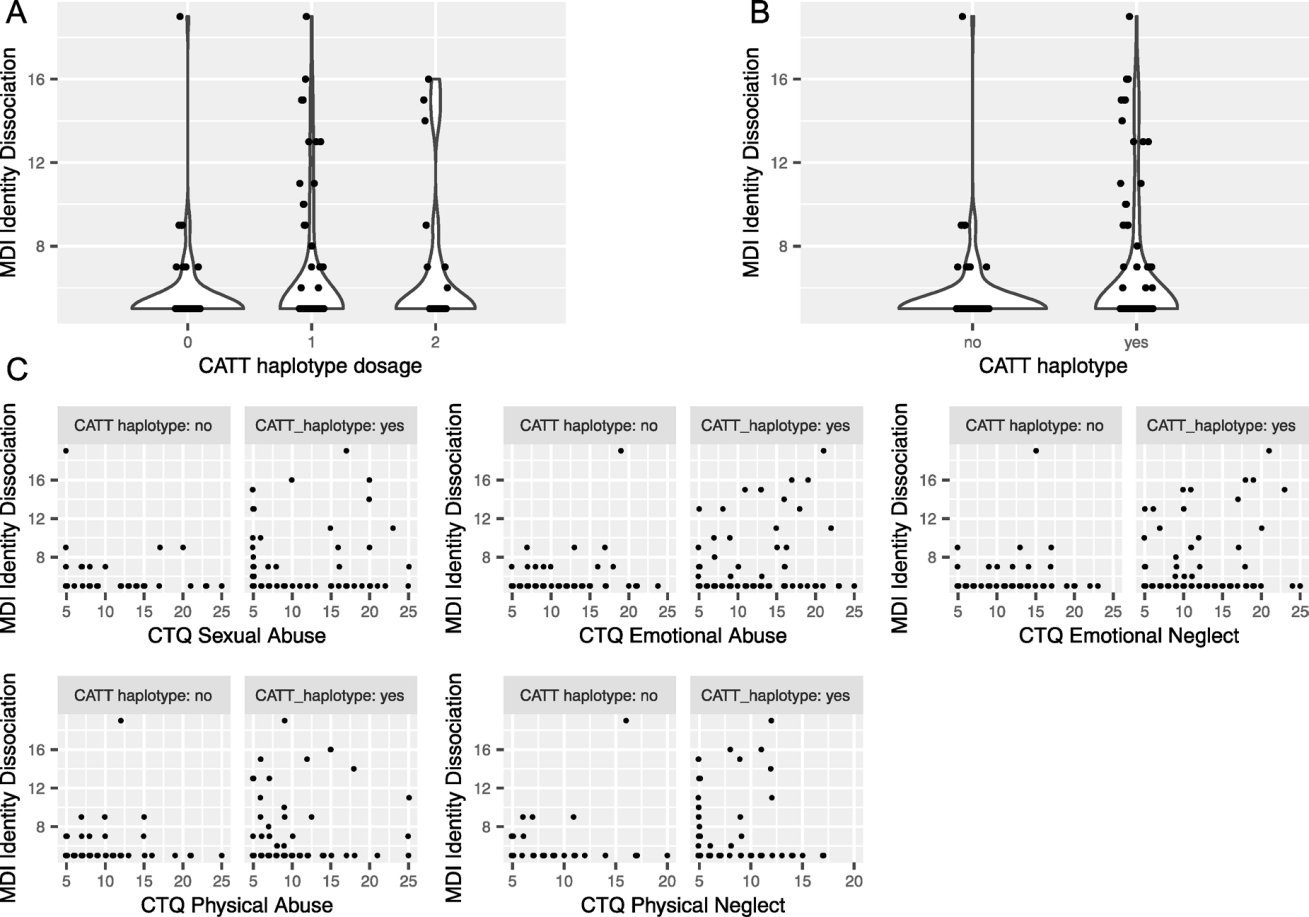
Association of individual dissociation subscales with the *FKBP5* CATT haplotype (**A**) Distribution of the MDI identity dissociation subscales in function of the number of CATT haplotypes (haplotype dosage). (**B**) Distribution of the MDI identity dissociation subscales in function of the presence or absence of at least one CATT haplotype (haplotype factor). (**C**) Interactions between CATT haplotype (as a factor) and different childhood stressors and the MDI identity dissociation score.

We assessed the association of the dissociative phenomena, measured with the Multiscale Dissociation Inventory (MDI), with the CATT haplotypes of the *FKBP5* gene, formed by 4 SNPs (rs3800373, rs9296158, rs1360780, and rs9470080). We performed regression tests with the continuous or binary MDI subscales as the outcome and the CATT haplotype dosage (Fig. 1A) or haplotype (Fig. 1B) as predictor. We found a significant association of the CATT haplotype with identity dissociation (Table 1). In particular, individuals harboring the CATT haplotype are 15 times more likely to develop identity dissociation than individuals with other FKBP5 haplotypes (Log OR = 2.952, RR = 15.625, and p-value = 0.093). Moreover, this association was significant as well when we considered the CATT haplotypes as dosage (log OR = 1.301 and adjusted p-value [Benjamini-Hochberg] = 0.037), indicating that homozygous individuals for the CATT haplotypes bear more risk than heterozygous. On a continuous scale, the CATT haplotype is associated with increasing strength of identity dissociation (beta = 0.246 and p-value = 0.093 for CATT factor, beta = 0.2 and p-value = 0.052 for CATT dosage). There were no significant associations between the CATT haplotype and the other MDI subscales (Table 1).

**Table 1:** Association of MDI scales with the FKBP5 CATT haplotype results from the linear and logistic regression models in the association of individual dissociation subscales with the *FKBP5* CATT haplotype encoded as a factor (present/absent, top block) or dosage (number of haplotypes, bottom block). The coefficients represents beta of the linear model for continuous MDI subscales and the log odds ratio of the logistic model for binary subscales. Q-value were computed using the Benjamini-Hochberg procedure. Significant results (Q-value < 0.1) are highlighted in bold.

Association with CATT as factor			
MDI Scale	Coefficient CATT haplotype factor	P-value CATT haplotype factor	Q-value CATT haplotype factor
Total	0.159	0.261	0.457
Disengagement	0.290	0.037	0.171
Depersonalization	0.116	0.314	0.489
Derealization	0.064	0.623	0.727
Emotional constriction	0.021	0.877	0.877
Memory disturbance	0.068	0.617	0.727
**Identity dissociation**	**0.246**	**0.013**	**0.093**
Total binary	0.096	0.770	0.830
Disengagement binary	0.991	0.082	0.240
Depersonalization binary	0.835	0.086	0.240
Derealization binary	0.704	0.135	0.314
Emotional constriction_binary	0.211	0.612	0.727
Memory disturbance binary	0.512	0.241	0.457
**Identity dissociation binary**	**2.952**	**0.007**	**0.093**

Association with CATT as dosage			
MDI scale	Coefficient CATT haplotype dosage	P-value CATT haplotype dosage	Q-value CATT haplotype dosage
Total	0.105	0.328	0.563
Disengagement	0.172	0.101	0.316
Depersonalization	0.078	0.367	0.563
Derealization	0.025	0.798	0.901
Emotional constriction	0.039	0.699	0.889
Memory disturbance	0.018	0.862	0.901
**Identity dissociation**	**0.200**	**0.007**	**0.052**
Total binary	0.031	0.901	0.901
Disengagement binary	0.742	0.043	0.203
Depersonalization binary	0.514	0.113	0.316
Derealization binary	0.375	0.241	0.563
Emotional constriction_binary	0.285	0.347	0.563
Memory disturbance binary	0.257	0.402	0.563
**Identity dissociation binary**	**1.301**	**0.003**	**0.037**

Numerous previous studies have reported that *FKBP5* interacts with childhood stressors and trauma to modulate the risk of psychiatric disorders^1,2,50,51^. We therefore also investigated the presence of such interaction in our dataset using linear regression with an interaction term between the different stressors, represented by different subscales of the CTQ in our dataset, and the CATT haplotype. Although we report a significant main effect of emotional abuse, emotional neglect and physical abuse on identity dissociation along with the significant effect of the CATT haplotype, none of the interaction terms are significant (Table 2), irrespective of the coding (dosage or binary) of the CATT haplotype. This suggests that for any given amount of childhood trauma, the presence of the CATT haplotypes favors identity dissociation (Fig. 1C). Concomitantly, a stronger childhood trauma increases dissociation, with or without CATT haplotypes. It is worth noting that the standardized coefficient for the CATT haplotype and the 3 associated CTQ scales (Table 2) are of the same order, indicating an effect of comparable magnitude between the *FKBP5* genetic predisposition and the strength of childhood traumas. Similar results were obtained when considering the binary version of the identity dissociation variable in logistic models (Table 2), confirming that the significant results are not due to violation of the linear model assumption (even after attempting an inverse normal transformation, the identity dissociation variable was not normally distributed).

**Table 2:** Association of the MDI identity dissociation subscale with childhood trauma and the FKBP5 CATT haplotype results from the linear regression models testing the association of the continuous MDI identity dissociation subscale with different childhood traumas, the FKBP5 CATT haplotype encoded as a factor (present/absent, top block) or dosage (number of haplotypes, bottom block) and their interaction. The std.coefficient columns indicates the standardized coefficients from the model. These coefficients are independent of the scale of the variable and allow to compare the strength of the effect of different variables. Significant results (P-value < 0.05) are highlighted in bold.

Association with CATT as factor							
CTQ	Coefficient CATT	Coefficient CTQ	P-value CATT	P-value CTQ	P-value interaction	Std.coefficient CATT	Std.coefficient CTQ
Sexual abuse	0.068	−0.007	**0.017**	0.457	0.269	0.121	0.027
Emotional abuase	0.135	0.023	**0.027**	**0.003**	0.653	0.108	0.144
Emotional neglect	0.124	0.019	**0.022**	**0.009**	0.589	0.112	0.127
Physical abuse	0.126	0.010	**0.020**	**0.132**	0.585	0.115	0.073
Physical neglect	0.236	0.011	**0.013**	0.463	0.957	0.123	0.037

Association with CATT as dosage							
CTQ	Coefficient CATT	Coefficient CTQ	P-value CATT	P-value CTQ	P-value interaction	Std.coefficient CATT	Std.coefficient CTQ
Sexual abuse	0.074	−0.004	**0.010**	0.473	0.303	0.127	0.027
Emotional abuse	0.154	0.026	**0.019**	**0.004**	0.887	0.114	0.141
Emotional neglect	0.171	0.024	**0.020**	**0.015**	0.991	0.114	0.122
Physical abuse	0.084	0.007	**0.014**	0.180	0.449	0.117	0.060
Physical neglect	0.201	0.011	**0.008**	0.516	0.996	0.132	0.032

## Discussion

To contribute to the large body of evidence linking *FKBP5* to several psychiatric disorders (for a review see^7^), we tested for associations between the CATT haplotypes of *FKBP5*, different types of childhood trauma, and different types of dissociative phenomena. The literature on genetic studies investigating dissociation is limited and has rarely examined the different types of dissociative phenomena (for a review, see^7,36–38^).

In our study, for detachment dissociation: disengagement, emotional constriction, depersonalization and derealization, and for compartmentalization dissociation: memory disturbance^9,52^ were not significantly associated with the CATT haplotype.

Only identity dissociation was significantly associated with the CATT haplotype, whether we considered the CATT haplotype as a dosage or as a factor. This association did not depend on the type or strength of childhood trauma. This suggests that for any given amount of childhood trauma, the presence of the CATT haplotype favors identity dissociation. The results are partly consistent with a study by Dackis et al. who found an association between the CATT haplotype, childhood maltreatment, and dissociation in a comparable population. However, in this study, dissociation was measured with the DES and only the total score was used for analysis^40^. In partial contradiction to our findings, Yaylaci and colleagues reported “a significant interactive effect of time/chronicity of maltreatment and CATT haplotype on dissociative symptoms”. Thus, they found increased dissociative symptomatology in combination with an absence of the CATT haplotype, chronic maltreatment and infancy onset of traumatization^39^. This discrepancy may be explained by the temporal resolution of their analysis, which could not be matched in this study. In addition, the adolescent DES was used to assess dissociation and only the total score was used for analysis^39^. Comparability is also limited by the fact that both studies did not examine different types of dissociative phenomena and did not include identity dissociation.

Because detachment dissociation and memory impairment are common in stress-related disorders and across all life stages, we suggest that these constructs may not be specific enough to detect gene-environment interactions early in development, which may explain our findings and is consistent with the inconsistent results of the few previous studies that have examined depersonalization/derealization symptoms as different types of dissociative phenomena^7^. In contrast, identity dissociation is thought to be associated with chronic and severe traumatic stress early in development^53,54^, and consistent with this, we found a significant association with the CATT haplotype, which may make identity dissociation an interesting phenomenon for further gene × environment studies.

From a developmental perspective, our findings add evidence to previous findings by Halldorsdottir and colleagues, who found that the CATT haplotype associated with early traumatic stress leads to developmental disturbances of stress regulation and cognitive function^55^. Due to our cross-sectional design, the results do not allow for a causal interpretation, but it appears that identity development could be disrupted by the same combination of childhood traumatic stress, regardless of the type of trauma, and the CATT haplotype.

Taking a more holistic view, our findings may help to understand how genetics and self-development interact. It appears that the CATT haplotype in combination with early traumatic stress leads to greater stress vulnerability with disruption of the HPA-axis^56^. This, in turn, appears to be associated with a disturbance in psychological development and, consistent with our findings, long-term disruption of identity development. Since identity dissociation is one of the most severe pathological dissociative phenomena and is often accompanied by other psychopathological symptom complexes^53,57^, the association found with genetic variants involved in stress systems is further evidence for the trauma model of dissociation and possibly a point of approximation of the complex relationships to disintegration models of trauma-induced dissociation between higher and lower mental functions^5,58^.

*FKBP5* is an important endogenous regulator of the stress response system, and genetic variations have been associated with many stress-related disorders, such as maladaptive emotional behavior, posttraumatic stress disorder, anxiety disorders and borderline personality disorder^1,56,59–62^. *FKBP5* influences many pathways involved in the stress response to adverse life events, such as HPA axis activation, cell proliferation, synaptic plasticity, autophagy or the autoimmune system^1,56^. For example, increased expression of *FKBP5* has been associated with increased anxiety, impaired extinction learning and reduced stress coping, which may be a pathway to stress-related psychopathology following early traumatic life events, particularly in high expression phenotypes^1,55,63^.

From an evolutionary perspective, variation in *FKBP5* may lead to better adaptation to a dangerous environment that requires a constant response with survival strategies^64,65^. Overactivation, in turn, may have long-term negative effects on the development of a reflective self and the subjective experience of phenomena such as identity dissociation^5^. This may be important for exploring the interplay between genes, environment, and psychopathology in disorders with a high prevalence of childhood adversity and developmental disorders of the self, such as borderline personality disorder or complex posttraumatic stress disorder^66,67^.

The timing and chronicity of trauma appear to be important for this relationship and for the development of severe dissociation^54,68,69^. Although we did not assess timing, we did use a self-report questionnaire specifically about childhood trauma. The importance of timing, chronicity of trauma and type of dissociation studied may also explain the inconsistent results of studies investigating the molecular basis of dissociation, as dissociative disorders, and in particular the different types of dissociation, have rarely been the focus of interest, and populations have not been homogeneous in terms of type and timing of trauma^7^.

Therefore, future studies should examine the relationships between *FKBP5* haplotype, timing of trauma and the different types of dissociation, including identity dissociation, in larger cohorts of trauma-related disorders, as well as other disorders associated with identity experience disturbance, such as borderline personality disorder and psychotic disorders. In psychotic disorders, there is considerable evidence for a transdiagnostic relationship between trauma, dissociation and positive psychotic symptoms^70^. Knowing more about the molecular basis of dissociation, could help identifying biomarkers for dissociative positive symptoms^36,37^, which would be helpful for differential diagnosis between psychotic disorders and trauma related disorders with psychotic symptoms as well as dissociative psychotic phenomenology in psychotic disorders.

### Limitations

Due to our cross-sectional design, the results do not allow for a causal interpretation. In addition to the absence of information regarding the occurrence time of the stressors, our study needs to acknowledge its sample size as a major limitation. Indeed, while the original size of the cohort with 1026 individuals could offer good power, this number dropped to 194 for the FKBP5 haplotype analysis because of missing phenotypic data. We computed the minimum detectible effect for a simple logistic regression model without covariate and for a dependent variable distributed similarly to the MDI subscales. With our sample size, the minimum detectible effect at a power of 80% with alpha 0.05 is 2.1. Although we did use models with covariates, and have likely not exactly the same power as using a simpler model, we argue that we had enough power to detect relevant effects. However, replication of the present results in a larger cohort would therefore be needed to further validate our findings.

In addition, generalizability is very limited because our sample consisted mainly of a highly traumatized urban sample of Black American women. Thus, the findings cannot be easily generalized to other ethnic groups or populations. In terms of type of trauma, we investigated childhood trauma, but the majority of this population is likely to have been exposed to a variety of traumatic experiences throughout their lives, which may have influenced the results of dissociative phenomena. Self-report of memories of childhood trauma and dissociative experiences may also be influenced by the current mental state of the individual.

Furthermore, it is likely, for example, that a relevant gender-specific aspect of the response to severe trauma could not be addressed due to study population. Relatedly, examination of other relevant stressors (e.g., racism, sexism) should be included in future studies examining gene × environment interactions in marginalized populations.

## Data Availability

The datasets used and/or analysed during the current study are available from Abigail Powers Lott (Co-Director, Grady Trauma Project) on reasonable request.
